# Comparative Genomic and Sequence Analysis Provides Insight into the Molecular Functionality of NOD1 and NOD2

**DOI:** 10.3389/fimmu.2013.00317

**Published:** 2013-10-07

**Authors:** Joseph P. Boyle, Sophie Mayle, Rhiannon Parkhouse, Tom P. Monie

**Affiliations:** ^1^Department of Biochemistry, University of Cambridge, Cambridge, UK

**Keywords:** NLR, NOD1/NOD2, comparative biology, evolutionary tracing, CARD, innate immunity, LRR

## Abstract

Amino acids with functional or key structural roles display higher degrees of conservation through evolution. The comparative analysis of protein sequences from multiple species and/or between homologous proteins can be highly informative in the identification of key structural and functional residues. Residues which in turn provide insight into the molecular mechanisms of protein function. We have explored the genomic and amino acid conservation of the prototypic innate immune genes NOD1 and NOD2. NOD1 orthologs were found in all vertebrate species analyzed, whilst NOD2 was absent from the genomes of avian, reptilian and amphibian species. Evolutionary trace analysis was used to identify highly conserved regions of NOD1 and NOD2 across multiple species. Consistent with the known functions of NOD1 and NOD2 highly conserved patches were identified that matched the Walker A and B motifs and provided interaction surfaces for the adaptor protein RIP2. Other patches of high conservation reflect key structural functions as predicted by homology models. In addition, the pattern of residue conservation within the leucine-rich repeat (LRR) region of NOD1 and NOD2 is indicative of a conserved mechanism of ligand recognition involving the concave surface of the LRRs.

## Introduction

NOD1 and NOD2 are prototypical members of the NLR family of cytosolic pattern recognition receptors and the human and murine proteins have been widely studied. Both receptors respond to different fragments of bacterial peptidoglycan, most likely through direct binding (1–3) although further confirmation of this is required (4). In the absence of ligand the C-terminal leucine-rich repeat (LRR) region contributes to autoinhibition, a state maintained by interaction with chaperone proteins including Hsp90 and SGT1 (5, 6). Exposure to ligand results in conformational rearrangement that permits receptor self-association and nucleotide binding via highly conserved amino acid motifs in the central NOD (or NACHT) region (7). This is coupled with migration to the plasma membrane and caspase activation and recruitment domain (CARD) mediated interaction with the adaptor protein RIP2 and/or CARD9. The net effect is to initiate a pro-inflammatory response mediated by NFκB and stress-kinase activated genes.

The functionality of NOD1 and NOD2 has been well characterized. Despite this we still have a limited understanding of the molecular basis of receptor function. At the amino acid level: changes in NOD2 can lead to an increased susceptibility to inflammatory disorders such as Crohn’s disease or cause conditions like Blau Syndrome (8–10); variation in the LRR of NOD1 explains the preferential recognition of tripeptide and tetrapeptide diaminopimelic acid containing peptidoglycan fragments by human and murine NOD1 respectively (11, 12); the C-terminus of NOD2 is important for membrane localization (13); and that specific patches are involved in RIP2 interaction (14, 15), Ubiquitin binding (16), and nucleotide binding and hydrolysis (7).

Amino acids that show high levels of conservation across multiple orthologs or homologs are indicative of residues with important structural or functional roles (17). Consequently comparative sequence analysis can be highly informative in the identification of functionally important residues. We have compared the amino acid sequences of NOD1 and NOD2 across vertebrate species in order to gain a greater understanding of the key functional regions of both proteins. Key functional patches, for example those involved in RIP2 binding and nucleotide binding and hydrolysis show strong, or even complete, conservation across species. Recognition of ligand is likely to be mechanistically conserved between both NOD1 and NOD2 and located on the concave surface of the LRR region and we provide further evidence for the importance of the C-terminus of NOD1 and NOD2 in the function and localization of the receptor.

## Materials and Methods

### Bioinformatics, database searching, and evolutionary tracing

The reference sequences for human NOD1 (NP_006083.1) and human NOD2 (NP_071445.1) were used as search terms to retrieve orthologous protein sequences from the NCBI protein database. Sequences with at least 95% sequence coverage were retained and collated in FASTA format. Sequences were aligned using MUSCLE (18) and then manually refined to remove incomplete and partial sequences. The resulting alignments were subjected to evolutionary tracing using TraceSuite II (19). Consensus sequence images were generated using WebLogo v3.3 (20). NetSurfP was used to predict the surface accessibility of individual amino acids (21). All molecular structure images were created using the PyMOL Molecular Graphics System, v1.5.0.5 Schrödinger, LLC.

To perform a pairwise comparison between the eight terminal LRRs in NOD1 and NOD2 we manually identified the relevant LRR sequences from the human, chimpanzee, mouse, cow, elephant, platypus, and coelacanth proteins. The number of identical residues between each possible pair of repeats where one repeat is from NOD1 and one repeat is from NOD2 was determined and these values averaged. The average values were tabulated and color coded on a sliding scale from green (most similar) to red (least similar).

### Homology modeling

Homology models were built using Modeller v9.8 with the following templates: NOD2 CARD1 – ICEBERG CARD [1DGN; (22)]; NOD2 CARD1– NOD1 CARD (2DBD); NOD1 and NOD2 LRRs – porcine ribonuclease inhibitor LRRs [2BNH; (23)]. Models were refined and the stereochemistry verified using PROCHECK (24).

### Plasmids

pUNO-NOD1 and pCMV-NOD2 (25) produce full-length untagged NOD1 and an N-terminally FLAG tagged NOD2 respectively; pLuc and phrG (Promega) encode Firefly and Renilla luciferase. Mutant constructs were generated by site-directed mutagenesis.

### Luciferase reporter assays

HEK293 cells were maintained in DMEM (Sigma) supplemented with 10% FCS, 100 μg/ml Penicillin/Streptomycin and 2 mM l-glutamine at 37°C and 5% CO_2_. Assays were performed in 96-well plates and using jetPEI (Polyplus Transfection) cells were transfected with 0.1 ng of NOD1/2 DNA and 1 ng of pLuc and phrG in each well. Cells were stimulated with specified concentrations of iE-DAP, muramyl dipeptide, or iE-Lys (all Invivogen), concomitant with DNA transfection. Cells were lysed 24 h post transfection with 1 × passive lysis buffer (Promega) and luminescence measured with a LUMIstar Luminometer (BMG Labtech). Protein expression was checked 24 h after transfection of HEK293 cells with 3 μg/DNA per well in a six-well plate without ligand stimulation. Proteins were detected with either monoclonal anti-FLAG (Sigma) or the NOD1 monoclonal 2A10 (26).

### Subcellular fractionation

Membrane and cytosolic fractionation of transfected HEK293 cells was performed using a Subcellular Fractionation Kit (Pierce) as per the manufacturer’s instructions. An antibody against GAPDH (Abcam) was used to characterize cytosolic fractions.

## Results

### NOD1 and NOD2 possess different evolutionary patterns

**Figure 1:**
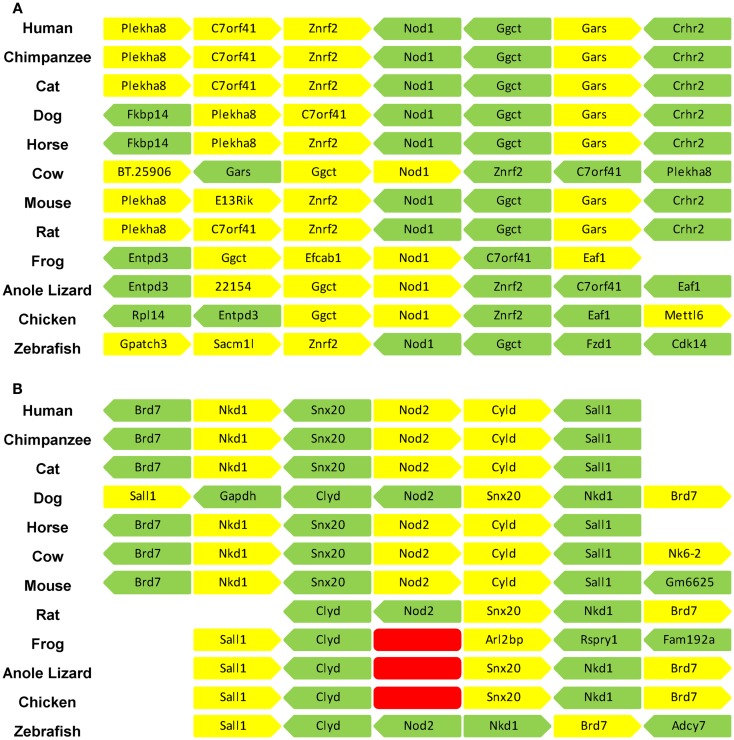
**The syntenic positions of *Nod1* and *Nod2* are highly conserved**. The syntenic position of **(A)** *Nod1* and **(B)** *Nod2* were compared in 12 different vertebrate species. The three adjacent genes upstream and downstream of *Nod1/2* are displayed. Each gene is represented by an individual block with yellow denoting a position on the forward strand and green a position on the reverse strand. A space indicates insufficient information to definitively identify the gene in that location. The red blocks in **(B)** indicate the absence of the *Nod2* gene from the frog, anole lizard, and chicken genomes. Gene identities are as follows: *Plekha8* – pleckstrin homology domain containing, family A (phosphoinositide binding specific) member 8; *C7orf41* – chromosome seven open reading frame 41; *znrf2* – zinc and ring finger 2; *Nod1* – nucleotide-binding oligomerization domain containing 1; *Ggct* – gamma-glutamylcyclotransferase; *Gars* – glycyl-tRNA synthetase; *Crhr2* – corticotrophin-releasing hormone receptor 2; *Fkbp14* – FK506 binding protein 14; *BT.25096* – corticotrophin-releasing factor receptor 2; *E13Rik* – RIKEN cDNA 241066E13 gene; *Entpd3* – ectonucleoside triphosphate diphosphohydrolase 3; Efcab1 – EF-hand calcium binding protein; *Eaf1* – ELL-associated factor 1-like; *Rpl14* – ribosomal protein L14; *Mettl6* – methyltransferase-like protein 6; *Gpatch3* – G patch domain containing 3; *Sacm1l* – SAC1 suppressor of actin mutations 1-like; *Fzd1* – frizzled homolog 1; *Cdk14* – cyclin-dependent kinase 14; *Brd7* – bromodomain containing 7; *Nkd1* – naked cuticle homolog 1; *Snx20* – sorting nexin 20; *Nod2* – nucleotide-binding oligomerization domain containing 1; *Cyld* – ubiquitin carboxyl-terminal hydrolase; *Sall1* – sal-like 1; *Gapdh* – glyceraldehydes 3-phosphate dehydrogenase; *Nkx6-2* – uncharacterized protein; *Gm6625* – protein Gm6625; *Arl2bp* – ADP-ribosylation factor-like 2 binding protein; *Rspry1* – ring finger and SPRY domain containing 1; *Fam192a* – family with sequence similarity 192, member A; *Adcy7* – adenylate cyclase 7.

Orthologs of human NOD1 and NOD2 were retrieved from the NCBI protein database. NOD1 orthologs were found in a wide range of mammalian species as well as birds, amphibians, and fish. Consistent with previous reports NOD2 was widely present in mammals and fish, but absent from avian and amphibian genomes (27). No reptilian orthologs were recovered for either protein. Given the otherwise ubiquitous pattern of NOD1 possession across vertebrates we examined the genome of the reptilian anole lizard in release 71 of the ENSEMBL genome database. This approach successfully identified NOD1 in the anole lizard, but revealed no evidence of a NOD2 ortholog (Figure 1; Table 1). Comparing the syntenic positions of NOD1 and NOD2 in a range of vertebrates confirmed the absence of NOD2 from reptiles (Figure 1; Table 1).

**Table 1:** **Chromosomal position and ENSEMBL identifier for *Nod1* and *Nod2* across diverse vertebrate species; n.d., not described**.

Species	*Nod1*		*Nod2*	
	Chromosome	ENSEMBL ID	Chromosome	ENSEMBL ID
Human	7	ENSG00000106100	16	ENSG00000167207
Chimpanzee	7	ENSPTRG00000019040	16	ENSPTRG00000008106
Cat	A2	ENSFCAG00000012184	E2	ENSFCAG00000008505
Dog	14	ENSCAFG00000003074	2	ENSCAFG00000009818
Horse	4	ENSECAG00000013825	3	ENSECAG00000017005
Cow	4	ENSBTAG00000038235	18	ENSBTAG00000020936
Mouse	6	ENSMUSG00000038058	8	ENSMUSG00000055994
Rat	4	ENSRNOG00000010629	19	ENSRNOG00000014124
Frog	n.d.	ENSXETG00000022012	–	–
Anole lizard	6	ENSACAG00000002919	–	–
Chicken	2	ENSGALG00000011535	–	–
Zebrafish	16	ENSDARG00000036308	7	ENSDARG00000010756

A closer examination of the syntenic position of *Nod1* indicated that for all species investigated, except the frog, *Nod1* was located between *Znrf2* (zinc and ring finger 2) and *Ggct* (gamma-glutamylcyclotransferase). All three genes either side of *Nod1* are strongly conserved, particularly between mammals (Figure 1A). The syntenic position of *Nod2* showed even greater conservation across mammals, sharing positions with *Brd7* (bromodomain containing 7), *Nkd1* (naked cuticle homolog 1), *Snx20* (sorting nexin 20), *Cyld* (ubiquitin carboxyl-terminal hydrolase (sometimes referred to as cylindromatosis), and *Sall1* (sal-like 1). The syntenic position is maintained in zebrafish except that *Snx20* has been lost. The chicken and anole lizard retained the whole genomic cluster except for *Nod2*; whilst in the frog only *Sall1* and *Clyd* are located together (Figure 1B). Performing a whole genome BLAST search and screening the expression sequence tag database did not detect *Nod2* in any of these organisms, nor in the Zebra Finch or Turkey. This indicates that in birds, reptiles, and amphibians the *Nod2* gene has been specifically lost.

### Mapping key residues in NOD1 and NOD2 by cross-species comparisons

**Figure 2:**
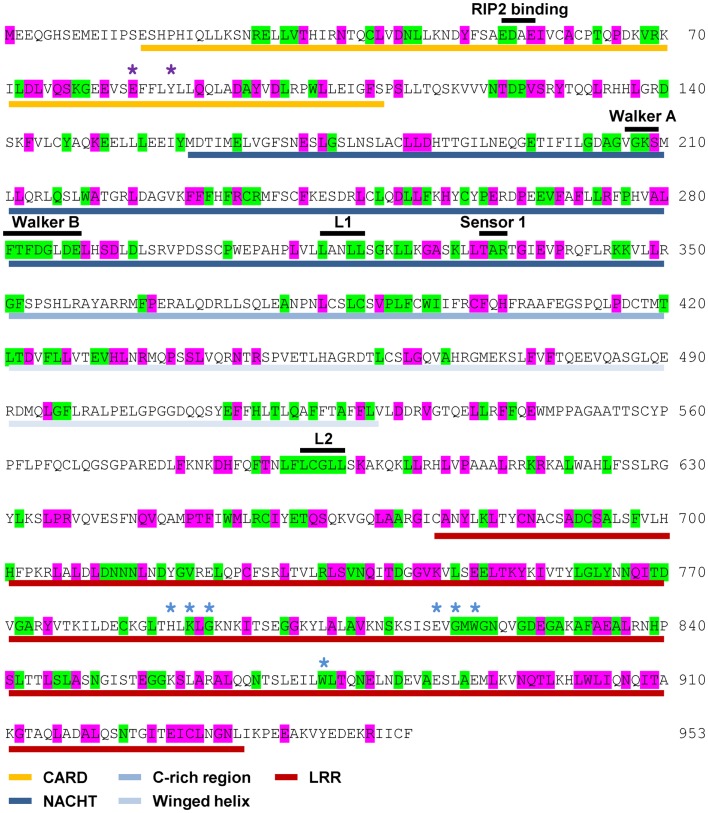
**Pattern of cross-species residue conservation in NOD1**. Residues conserved across all NOD1 species checked, or just across mammals, are highlighted green and purple respectively. Residues are mapped onto the amino acid sequence for human NOD1. The domain architecture is highlighted underneath the relevant stretch of sequence as follows: CARDs – gold; NACHT – dark blue; C-rich region – blue; Winged-helix – pale blue; LRRs – red. The motifs responsible for RIP2 binding, the Walker A and B motifs and the Sensor 1 region are all labeled in black above the relevant sequence. Also labeled above the sequence are LxxLL motifs [black bars (L1, L2)], residues predicted to be important for ubiquitin binding (purple asterisks), and residues predicted to be involved in ligand recognition (blue asterisks).

**Figure 3:**
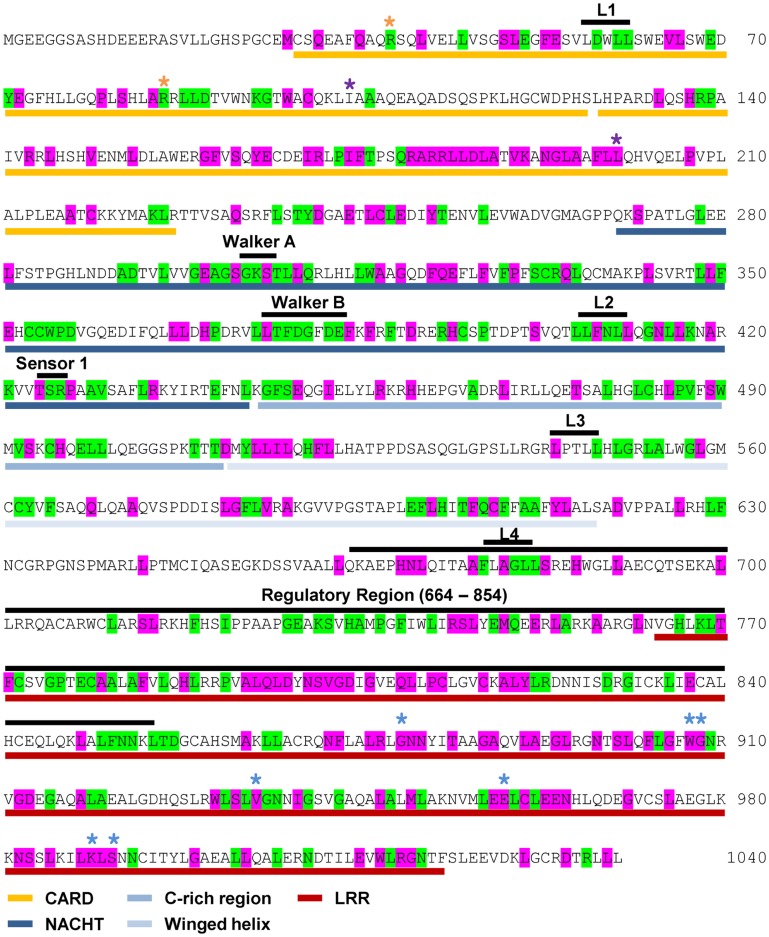
**Pattern of cross-species residue conservation in NOD2**. Residues conserved across all NOD2 species checked, or just across mammals, are highlighted green and purple respectively. Residues are mapped onto the amino acid sequence for human NOD2. The domain architecture is highlighted underneath the relevant stretch of sequence as follows: CARDs – gold; NACHT – dark blue; C-rich region – blue; Winged-helix – pale blue; LRRs – red. The motifs responsible for RIP2 binding, the Walker A and B motifs, the Sensor 1 region and the regulatory region from residues 664 to 854 are all labeled in black above the relevant sequence. Also labeled above the sequence are the LxxLL motifs [black bars (L1, L2, L3, L4)], residues predicted to be important for interaction with RIP2 (yellow asterisks), ubiquitin binding (purple asterisks), and residues predicted to be involved in ligand recognition (blue asterisks).

NOD1 and NOD2 amino acid sequences were aligned and evolutionary tracing was used to examine the amino acid conservation at two levels. The first level consisted of residues completely conserved across all vertebrate species. The second level represented residues completely conserved in mammals, but not across all of the non-mammalian sequences. The patterns of conservation are summarized on the human NOD1 (Figure 2) and NOD2 (Figure 3) amino acid sequences.

Levels of cross-species amino acid conservation were highly similar for NOD1 and NOD2 (Table 2). Conserved residues were broadly dispersed across both protein sequences with denser, more focused, patches seen in the CARD, NACHT, and LRR domains (Figures 2 and 3). These included motifs of known function such as the RIP2 binding patch in the NOD1 CARD; the Walker A, Walker B, and Sensor 1 motifs crucial for nucleotide binding and hydrolysis in the NACHT; and the LRR consensus repeats (7, 14, 28). In NOD2 the first 27 residues, the C-terminal region of CARD1 and also the linker portion between the end of the winged-helix domain and the start of the regulatory region showed particularly low patterns of conservation (Figure 3). The second CARD of NOD2 and three sections of the NOD2 LRRs – A794-Y821, N872-F903, E962-S991 – showed strong conservation across mammals, but not when piscine NOD2 was included.

**Table 2:** **Levels of cross-species amino acid identity for NOD1 and NOD2**.

	Percentage of identical amino acids	
	All species	Mammalian sequences
NOD1	19.3	39.8
NOD2	18.3	37.0

NOD1 and NOD2 show varying degrees of conservation of the protein interaction motif LxxLL, a motif commonly found in nuclear receptors. Two LxxLL motifs, beginning at L314 and L592, are completely conserved across all species of NOD1 (Figure 2). Human NOD2 contains four LxxLL motifs starting at residues L57, L407, L554, and L678. The second of these, L_407_xxLL, is in the NACHT domain and correlates with the NOD1 motif beginning at L314. Unlike NOD1, none of the NOD2 LxxLL motifs are completely conserved across all species. However, L_407_FNLL is conserved across mammals.

### Different biological processes merit different levels of residue conservation

**Figure 4:**
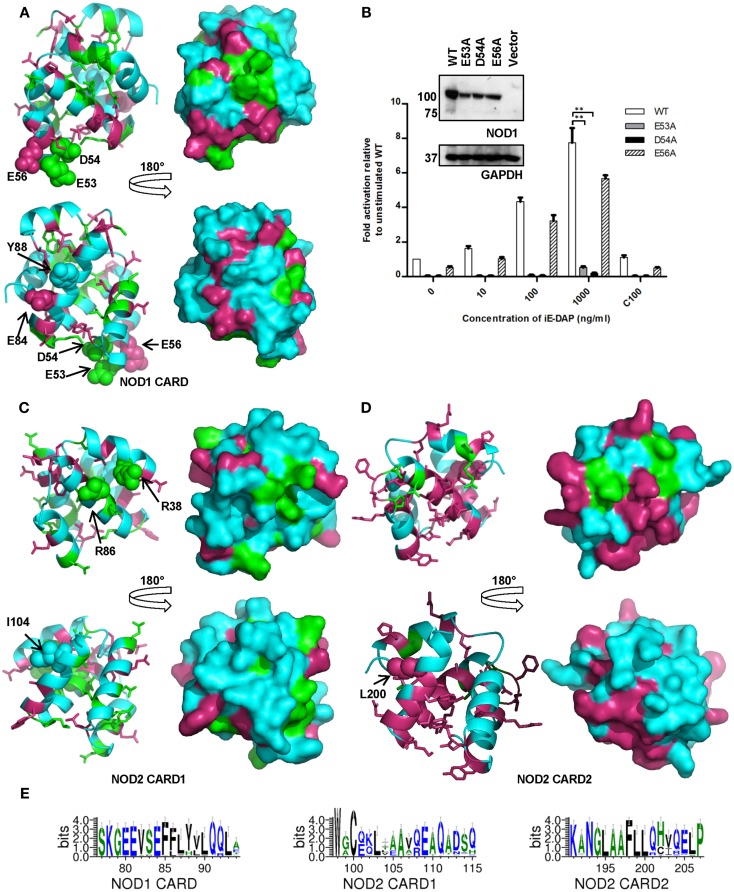
**Amino acid conservation in the NOD1 and NOD2 CARDs**. Cartoon and surface representations of NOD1 CARD **(A)**, NOD2 CARD1 **(C)**, and NOD2 CARD2 **(D)** showing amino acids conserved across all species (green) and conserved across mammals (pink). In each panel the top and bottom images are related by a 180° rotation around the vertical axis. The left and right images are cartoon and surface representations of the same view respectively. Residues previously implicated in interaction with RIP2 (NOD1 – E53, D54, E56; NOD2 – R38, R36) or in the process of ubiquitination (NOD1 – E84, Y88; NOD2 – I104, L200) are labeled and presented as spheres. Conservation is mapped onto an experimental NOD1 structure (PDB ID: 2DBD) and homology models of the NOD2 CARDs. **(B)** Differential contributions to receptor activation. NFκB luciferase reporter assays were performed in HEK293 cells using wild-type (WT) NOD1, E53A, D54A, and E56A constructs. DNA (0.1 ng/well) and varying concentrations of stimulatory (i.e., DAP) or control (i.e., Lys) ligands were transfected into 96-well plates. After 24 h cells were lysed and NFκB activity determined. Results show the average of four independent experiments and **p < 0.005. Error bars indicate SEM. Immunoblots (1.5 μg DNA/well in a six-well plate) were lysed after 24 h and probed with the specified antibodies to determine expression levels of NOD1 WT and mutant constructs. Immunoblots are representative of at least three separate experiments. **(E)** Patterns of conservation in the primary sequence observed around residues implicated in the ubiquitination of the CARDs. Residues are colored according to hydrophobicity (green – hydrophobic; blue – hydrophilic). Sequence images were generated using WebLogo 3.3 (20).

Manon and colleagues (14) previously identified acidic residues in the NOD1 CARD (E53, D54, E56) as crucial for interaction with RIP2. E53 and E54 are completely conserved across all species (Figures 2 and 4A), whereas E56 is completely conserved only in mammals. Closer inspection of the individual sequences shows that only the fish *Takifugu rubripes* differs at this position, possessing a highly conservative aspartic acid substitution. The role of these residues in NOD1 signaling was previously investigated using charge-reversal mutations (14). In order to avoid the potential influence of charge-repulsion effects due to the introduction of a positive charge we instead mutated each residue to alanine and tested their ability to activate NFκB-mediated signaling in response to ligand stimulation. The critical nature of E53 and D54 for NOD1 function was confirmed by the inability of either E53A or D54A to respond to ligand stimulation. E56A activity however did not differ significantly from the wild-type (Figure 4B). The slight reduction observed is likely due to the marginally lower expression of E56A compared to wild-type NOD1 (Figure 4B inset). Consequently, the impaired signaling of E56K and also its failure to interact with RIP2 (14) may be due to electrostatic repulsion, rather than indicating a critical role for E56 in RIP2 binding and NOD1 signaling.

For NOD2 two arginine residues, R38 and R86 in CARD1, are implicated in the interaction with RIP2 (15). These residues are completely conserved consistent with a crucial role in NOD2 function (Figure 3). We mapped R38 and R86, as well as the other completely conserved residues, onto a homology model of NOD2 CARD1 to determine if they associated to the same molecular surface. R38 and R86 were adjacent to each other and clustered with the surface-exposed residues D90 and K95, suggesting the possibility of larger electrostatic interface (Figure 4C). L89, which forms part of the hydrophobic core, also clustered to this region. There were fewer conserved residues in NOD2 CARD2 and these predominantly clustered to the helix 2-helix 3 loop, helix 3, and the helix 3-helix 4 loop (Figure 4D).

Ubiquitination is important in immune signaling. It regulates RIG-I signaling (29, 30) and is implicated in regulation of RIP2 signaling (31–33). Recently a competitive interaction between RIP2 and ubiquitin for binding to the NOD1 and NOD2 CARDs has been reported (16) with E84 and Y88 in NOD1 and I104 and L200 in NOD2 implicated as important for ubiquitin binding. E84 is completely conserved in mammals (Figures 2 and 4E) and only differs in five species of fish in which it is mutated to an alanine. Y88 is less well conserved, although most substitutions are for other bulky residues such as phenylalanine and histidine (Figure 4E). I104 and L200 occupy almost identical positions in the first and second CARD of NOD2. However, whilst L200 is completely conserved across mammals, I104 is often substituted for another hydrophobic residue (Figure 4E). Mapping these residues on to the structure of the NOD1 CARD and our models of the NOD2 CARDs indicated that neither NOD2 I104 nor L200 are as exposed on the molecular surface as E84 and Y88 are in NOD1 (Figures 4A–C). We validated this observation using NetSurfP which predicted that NOD1 E84 and Y88 are surface-exposed, but that NOD2 I104 and L200 are buried.

### Conservation in the LRRs provides insight into ligand binding

**Figure 5:**
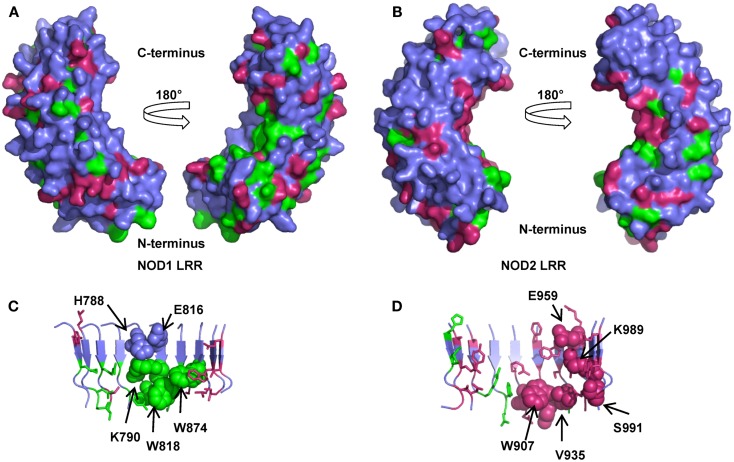
**Amino acid conservation in the NOD1 and NOD2 LRRs**. Surface representations of homology models of the NOD1 **(A)** and NOD2 LRRs **(B)** showing residues conserved across species (green) or just across mammals (pink). For clarity signature residues conserved in the consensus LRR repeat LxxLxLxxNxL (signature residues L and N) are not represented. The left and right images in **(A,B)** are related by a 180° rotation around the vertical axis. Cartoon representations of the concave surface of the NOD1 **(C)** and NOD2 **(D)** LRRs highlight the spatial relationships of residues likely to be involved in ligand detection. The side chains of residues previously implicated in ligand detection and receptor activation are represented as spheres and labeled appropriately except for G792, G818 (NOD1), and G879, G908 (NOD2). Residues are colored as for **(A,B)**.

Consistent with the repeating modular nature of the LRRs both NOD1 and NOD2 show increased conservation in this region. This is greatest around the consensus LRR motif LxxLxLxxNxL (where L = Leu, Val, Ile, Phe; N = Asn, Cys, Ser, Thr; x = any amino acid; signature residues underlined) (Figures 2 and 3). We mapped the completely conserved and mammalian-conserved residues onto homology models of their respective LRR regions but chose not to annotate any of signature residues to allow a focus on functional importance (Figure 5). Both NOD1 and NOD2 show molecular surfaces more conserved toward the N-terminus of the LRRs and on a single lateral surface (Figures 5A,B).

Mutagenic studies have identified regions of the LRR important for receptor activation (Table 3) (12, 34, 35). Five of the seven NOD1 residues are completely conserved across all species (Figures 2 and 5C). H788 is predominantly found as a histidine in mammals except for the pig where it is a cysteine and the horse, elephant, West Indian manatee, Northern greater galago, nine-banded armadillo, and white rhinoceros in which it is a tyrosine. In the non-mammalian species this residue is substituted by threonine, arginine, valine, and isoleucine. E816 has previously been implicated in selectivity for preferential activation by ligands with either tripeptide or tetrapeptide stems (11, 12). Consistent with a role in selectivity this residue was found as either an aspartic acid (26/53 sequences) or a glutamic acid (27/53 residues). All seven residues previously implicated in NOD2 activation are conserved across mammals, but not other species (Figures 3 and 5D). In the case of K989 and S991 this is due to the lack of a single LRR-encoding exon in Actinopterygii orthologs. Mapping these residues to the predicted structures revealed clustering around the edges of the concave surface of the LRR for both NOD1 and NOD2 (Figures 5C,D). When all conserved residues are considered the interface extends around the whole concave surface. These residues routinely appear in the second, third, fourth, and to a lesser extent fifth, variable positions in the consensus LRR motif (Lx_1_x_2_Lx_3_Lx_4_x_5_Nx_6_L) providing further support for a crucial functional role (Table 3).

**Table 3:** **Residues contributing to potential ligand binding patches on NOD1 and NOD2**.

	Residues previously implicated by mutagenesis^a^	Conserved residues with a potential to form part of a ligand binding interface
NOD1	H788, K790, G792, E816, G818, W820, W874	Y679, L706, D711, N712, R734, S736, V737, I757, G762, Y764, G821, S846, A848, T876, T897, W902, I904, E928, C930, G933
NOD2	G879, W907, G908, V935, E959, K989, S991	H766, K768, T770, A794, Q796, D798, A819, Y821, R823, F851, N852, R877, N880, F903, G905, W931, S933, G936, E958, C960, E962, E963, E1015, W1017

^a^ From (12, 35).

**Figure 6:**
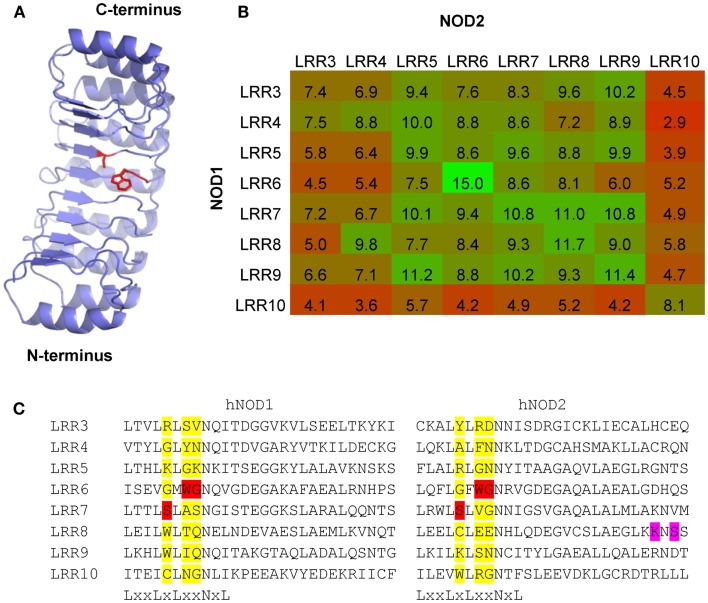
**Patterns of LRR conservation between NOD1 and NOD2 support a conserved ligand binding surface**. **(A)** Other than the signature residues in the LxxLxLxxNxL motif only three residues – W820, G821, and S846 (NOD1); W907, G908, and S933 (NOD2) – are conserved across all examined species of NOD1 and all mammalian NOD2 sequences. The likely spatial position of these residues on the concave surface of NOD1 is shown. The residue sidechains are represented as red sticks. **(B)** Heat map representation of the relative similarity of the eight terminal LRR repeats in NOD1 and NOD2 from the human, chimpanzee, mouse, cow, elephant, platypus, and coelacanth. The number in each box represents the average number of identical residues in a cross-species pairwise comparison between the relevant LRR motifs. Boxes are colored on a graded scale from green (most similar) down to red (least similar). **(C)** The three residues (highlighted red) are found in the X3, X4, and X5 position of the LRR consensus motif. These positions are populated by a wide range of different amino acids (highlighted yellow). K989 and S991 (highlighted in purple), two residues in human NOD2 implicated in ligand recognition and receptor activation, are located in a region of the protein missing in the Actinopterygii due to an exon deletion.

The similar patterns of residue conservation on the concave surface of NOD1 and NOD2, and the chemical similarities in activatory ligand, led us to ask exactly how alike these regions of the two proteins are. To begin we compared the eight terminal LRRs (LRRs 3–10) from human NOD1 and NOD2 to identify identical residues on the concave surface. Apart from the LxxLxLxxNxL motif, only seven identical residues were found and only three of these – W820, G821, and S846 (NOD1); W907, G908, and S933 (NOD2) – were fully conserved in all examined species of NOD1 and all mammalian NOD2 sequences (Figures 2 and 3). Spatially these residues are predicted to be in close proximity and may form a binding site for the shared elements in NOD1 and NOD2 ligands (Figure 6A). In support of this possibility, the conserved glycine in NOD2 has been thoroughly investigated as a SNP (G908R) which predisposes to Crohn’s Disease and reduces the ability of NOD2 to respond to MDP. In addition, a W907L NOD2 mutant was generated by Tanabe et al. and was found to eliminate the response to NOD2 (35). The role of the conserved serine is yet to be investigated.

We accompanied the search for individual residues in the LRRs with a broad examination of repeat similarity. The eight terminal repeats are formed of 28 amino acids each, with the final repeat showing greater sequence divergence and possibly stabilizing the end of the domain in a similar way to LRR capping structures (36). We compared these eight LRRs from the human, chimpanzee, mouse, cow, elephant, platypus, and coelacanth in order to look for identical residues. LRR6 was more similar between NOD1 and NOD2 than any other set of repeats, presumably reflecting a conserved functional role (Figure 6B). This repeat contains the WG motif discussed above and the adjacent LRR7 contains the conserved serine. Comparison across LRRs show that none of these conserved residues are commonly found in this position in multiple repeats and so are unlikely to be structurally important to the domain fold (Figure 6C).

### Disruption of conserved C-terminal residues alters receptor signaling and membrane localization

**Figure 7:**
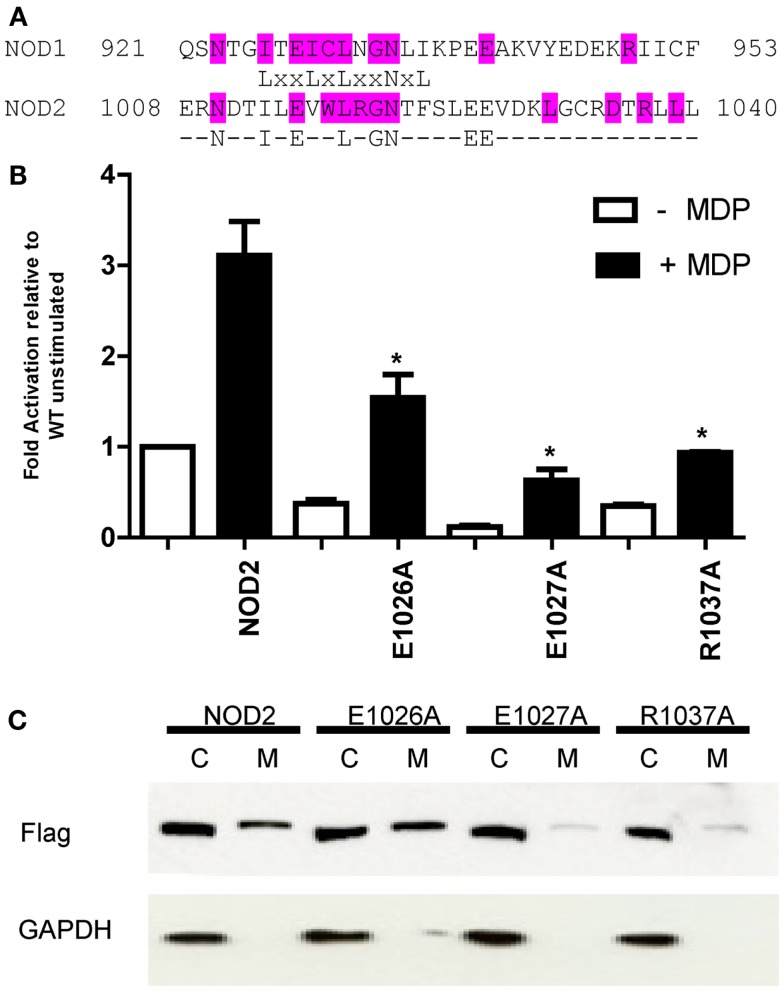
**The impact of mutation of conserved residues between the C-terminus of human NOD1 and NOD2 on receptor function**. **(A)** Alignment of the terminal 33 amino acids of human NOD1 and NOD2. Residues highlighted in cyan are conserved across mammals in the relevant protein. The consensus sequence highlights residues found in the termini of both human NOD1 and NOD2. **(B)** NFκB luciferase reporter assays were performed in HEK293 cells using wild-type pCMV-NOD2 and the point mutants E1026A, E1027A, and R1037A. DNA (0.1 ng/well) was transfected into 96-well plates with (black bars) and without (white bars) muramyl dipeptide (MDP). After 24 h cells were lysed and NFκB activity determined. Results show the average of three independent experiments and *p < 0.05. Error bars indicate SEM. **(C)** Subcellular fractionation was performed with wild-type and mutant NOD2 constructs to separate the cytoplasmic (C) and membrane-bound (M) fractions. Proteins were identified with the specified antibodies. Blots are representative of three independent experiments.

NOD1 and NOD2 are both targeted to the plasma membrane following activation (13, 26, 37, 38). The NOD2 1007fsincC Crohn’s Disease susceptibility polymorphism lacks the last 33 amino acids and doesn’t membrane localize (13). In fact the terminal three leucine residues appear important for localization. The final 33 amino acids of mammalian NOD1 and mammalian NOD2 show that the final LRR in both proteins is well conserved (Figure 7A). Outside this region residue conservation differs between the two proteins except that both human NOD1 and NOD2 have an EE motif starting 15 residues before the end of the protein, the second residue of which is conserved in mammalian NOD1 sequences. A closer examination of this motif showed that it is in fact highly conserved in NOD1 and NOD2 for most mammals. In NOD1 only the nine-banded armadillo varies in the first position, which is substituted for an aspartic acid. With NOD2 the EE motif is conserved in all mammalian sequences except the star-nosed mole in which the sequence is AD. In light of this degree of conservation we mutated both these residues, and also R1037 (conserved in NOD2 and immediately prior to the terminal LLL motif), in NOD2 to alanine and assessed the impact on receptor activation and protein localization following overexpression in HEK293 cells. All three mutants were significantly impaired in their ability to respond to muramyl dipeptide stimulation in comparison to the WT unstimulated protein (Figure 7B; *p*-values: E1026A = 0.042; E1027A = 0.025; R1037A = 0.029). However, each mutant also displayed a reduction in basal signaling in the absence of MDP. This resulted in the following approximate fold-increase in signaling for each construct: WT (threefold), E1026A (fourfold), E1027A (fivefold), and R1037A (threefold). As such, none of the mutants show impairment in their relative responses to ligand stimulation. Despite their ability to still respond to MDP neither E1027A nor R1037A were recruited to the plasma membrane (Figure 7C).

## Discussion

Comparative biology has the potential to rationalize and explain experimental observations and identify potentially key functional amino acids. We have performed, to our knowledge, the first comprehensive cross-species comparative analysis of the amino acid composition of NOD1 and NOD2. Reassuringly we found that regions of NOD1 and NOD2 already reported to provide essential functional roles showed increased, or even complete, conservation across species. Most notably these related to the Walker A and B motifs in the NACHT domain, the consensus region of the LRR motifs and residues crucial for interaction with the downstream adaptor protein RIP2 in NOD1 and NOD2.

Our analysis identified conserved LxxLL motifs in NOD1 and mammalian NOD2. LxxLL motifs are routinely used in nuclear receptors and form a key part of the nuclear receptor box (39). The precise function of the LxxLL motifs in NOD1 and NOD2 is currently unknown, however, it is highly unlikely that either NOD1 or NOD2 has an as yet unidentified nuclear role. The LxxLL motif has previously been reported in NACHT domains, including those of various plant R proteins which are divergently evolved relatives of the vertebrate NLR family (40, 41). In addition, oligomerization of the NLR protein CIITA utilizes an LxxLL motif in the NACHT domain (42). It is plausible that the conserved LxxLL motifs beginning at L314 (NOD1) and L407 (NOD2) provide a similar functionality.

The pattern of residue conservation in the LRRs of NOD1 and NOD2 is highly similar and points strongly toward a conserved mechanism of ligand binding and/or recognition on the concave surface of the LRRs. The mapping of these key residues to the concave surface is consistent with earlier work (12, 35), however, we have shown here that this interface may be more extensive than previously thought. In both NOD1 and NOD2 highly conserved residues increase the potential size of this interface and provide clear candidates for future mutagenesis studies.

Both NOD1 and NOD2 bind peptidoglycan fragments but discriminate between Lys-Type and diaminopimelic acid (DAP)-type muropeptides (34). This binding specificity is also seen in the Peptidoglycan Recognition Proteins (PGRPs), for which structural information has been used to identify the residues responsible for this difference in binding (43, 44). For NOD1 only the d-isoglutamyl-m-DAP moiety is required for signaling, but the presence of the preceding alanine enhances this response (34). In contrast, MDP, which consists of the MurNAc-l-alanine-d-isoglutamine segment, can signal effectively through NOD2. The similar ligands, and the similar patterns of conservation on the concave surface, suggest that the NOD1/2 ancestral gene could bind a muropeptide. Following gene duplication these binding sites evolved to permit the binding of distinct ligands by NOD1 and NOD2. We predict in NOD1 and NOD2 a mechanism similar to that of the PGRPs, where the comparable muropeptide ligands are bound in the same orientation but are told apart by their third peptide. An extra level of subtlety is displayed by the different species sensitivities of NOD1 to tripeptide and tetrapeptide stem lengths (11, 12). We have seen a clear split between the possession of either an aspartic acid or a glutamic acid residue in the equivalent position to human NOD1 E816. Indicating NOD1 has consistently evolved to respond preferentially to either tripeptide stem lengths (glutamic acid) or tetrapeptide stems (aspartic acid). Whether this is driven by exposure to particular microbiota remains unknown.

Unlike NOD1, NOD2 is not ubiquitously present in all species and the specific loss of the gene in birds, reptiles, and amphibians raises many questions about its evolutionary and functional roles. For example, what drives gene loss? Is this due to the absence of specific pathogenic threats in these populations? Interestingly multiple areas of NOD2 show strong conservation across mammals, but differ in the Actinopterygii orthologs. This is particularly noticeable in the LRRs. Actinopterygii orthologs of NOD2 are missing a single LRR-encoding exon which contains two residues which have been reported to contribute to the human MDP response, and which will alter the overall fold of the LRR. While the ability of these orthologs to respond to MDP or other muropeptides has not been investigated, it is possible in light of the NOD2 complete gene loss in birds, the anole lizard, and the frog that this function has also been lost in the actinopterygii.

The patterns of evolutionary conservation observed have increased the clarity of some functions, such as ligand-mediated activation, of NOD1 and NOD2. However, they have also raised questions of other published observations. Previously, Manon et al. reported that NOD1 E56 was essential for signaling as receptor activity was abrogated following mutation to lysine (14). The near-complete conservation of E56 across species supports an important functional role, however, mutating this residue to an alanine retains signaling, suggesting that at least some mutations are tolerated and that E56 is not absolutely critical for NOD1 signaling. Mutation of the acidic residues in the NOD1 EDAE motif will have reduced their spatial occupancy but their predominantly surface-exposed nature makes it unlikely that the native fold of the protein will have been perturbed (45). Our comparative analysis also suggests that the role of ubiquitin in NOD1 and NOD2 signaling may be more complex than previously imagined (16). The observed cross-species variation in NOD1 Y88 and NOD2 I104 suggests that the role of ubiquitin binding might differ between species; or that it is the general surface properties of this region, not the exact residues, that are important. Our homology modeling suggested that NOD2 I104 and L200 may be buried residues, mutation of which could disrupt the overall fold of the CARD. However, in the absence of a structure of the NOD2 CARDs this possibility remains theoretical and awaits experimental confirmation.

We identified a highly conserved di-acidic motif in the C-terminal region of both NOD1 and NOD2. Mutant NOD2 constructs showed reduced signaling compared to unstimulated wild-type NOD2, but also displayed a reduced basal level of activity. This resulted in the relative fold-increase for each construct being broadly comparable to wild-type NOD2. Despite their ability to still respond to MDP neither E1027A nor the downstream R1037A were efficiently recruited to the plasma membrane. Hence membrane recruitment may not be essential for NOD2 signaling, but might contribute to maximizing the efficiency of signaling. An earlier study by Barnich and colleagues (13) showed that a double EE to GG mutation did not alter membrane localization, or significantly disrupt NFκB signaling. Coupled with our data this raises the question of what the actual role of the EE motif is. Its high level of conservation suggests an important functional role, but this has yet to be experimentally confirmed and merits further investigation.

Overall, our work highlights the applicability of comparative biology and cross-species sequence analysis toward understanding the molecular basis of innate immune receptor function. It is an approach that if more widely used could provide extensive rewards in relation to efficiency savings by readily identifying suitable targets for functional study. Furthermore, it helps to provide a rationalization for the results of mutagenic studies thereby enabling an improved understanding of innate immune function.

## Conflict of Interest Statement

The authors declare that the research was conducted in the absence of any commercial or financial relationships that could be construed as a potential conflict of interest.
